# Evaluation of shedding, tissue burdens, and humoral immune response in goats after experimental challenge with the virulent *Brucella melitensis* strain 16M and the reduced virulence vaccine strain Rev. 1

**DOI:** 10.1371/journal.pone.0185823

**Published:** 2017-10-13

**Authors:** Jennifer L. Higgins, Mercedes Gonzalez-Juarrero, Richard A. Bowen

**Affiliations:** 1 Department of Microbiology, Immunology and Pathology, Colorado State University, Fort Collins, Colorado, United States of America; 2 Department of Biomedical Sciences, Colorado State University, Fort Collins, Colorado, United States of America; East Carolina University Brody School of Medicine, UNITED STATES

## Abstract

*Brucella melitensis* is the causative agent of brucellosis in small ruminants and is of considerable economic and public health importance in many countries worldwide. The control of disease in humans depends on the control of disease in livestock; however, few counties with endemic *B*. *melitensis* infection have been able to successfully eradicate this pathogen. This underscores the need for further research on the pathogenesis of both virulent and vaccine strains of *B*. *melitensis* in the small ruminant host. The aim of the present study was to characterize clinical effects, tissue colonization, shedding, and humoral immune response following *B*. *melitensis* infection in goats. Both virulent (16M) and reduced virulence (Rev. 1) strains of *B*. *melitensis* were studied. Pregnant goats were infected at 11–14 weeks of gestation with 8 x 10^6^ or 8 x 10^7^ CFU of *B*. *melitensis*. Infection of goats with *B*. *melitensis* 16M resulted in an 86% abortion rate. This strain disseminated widely in pregnant does post-infection with none of the 15 sampled tissues spared from colonization. Importantly, we report the first isolation of *B*. *melitensis* from muscle tissue in ruminants. Pathogenesis of Rev. 1 infection was variable with two does showing minimal colonization and one doe exhibiting disease similar to that of animals infected with fully virulent 16M. Shedding of *B*. *melitensis* in milk occurred in all 16M- and Rev. 1- infected goats. In pregnant animals challenged with virulent *B*. *melitensis*, median time to seroconversion was 21 days; however, 2 animals did not seroconvert until after abortion.

## Introduction

*Brucella melitensis*, a gram-negative bacterial pathogen, is the causative agent of brucellosis in small ruminants. This is an economically important disease endemic to many sheep and goat raising countries worldwide. Infection spreads rapidly among flocks causing abortion storms and chronic infection. While goats are considered the true natural hosts of *B*. *melitensis*, sheep are also highly susceptible [1]. In much of the Mediterranean basin and Middle East, *B*. *melitensis* is also the *Brucella* species most commonly isolated from bovine, camelid, and equine hosts [2].

Several *Brucella* species are highly pathogenic in humans, with *B*. *melitensis* typically cited as the agent responsible for the majority of human cases [3]. Brucellosis is one of the most common zoonotic diseases worldwide with over half a million new cases reported annually [4]. Infection most commonly results from ingestion of unpasteurized milk or contact with infected animals, and a chronic debilitating disease often develops if left untreated. Acute febrile illness can progress to focal disease affecting the joints, reproductive organs, nervous system, or rarely the heart [5–7].

*Brucella melitensis* was the first of the brucellae discovered, isolated from soldiers with Mediterranean fever on the island of Malta in 1887 and later from goats on the same island. Extensive research on the development of diagnostic tests and a suitable vaccine was pursued in Malta in the first half of the 20^th^ century. Control of caprine and ovine brucellosis is currently dependent on vaccination with *B*. *melitensis* strain Rev. 1 as well as test and slaughter programs. Rev. 1 is a live, attenuated strain with unknown mutations conferring attenuation. The vaccine was created in 1957 by sequential passage of a wild type *B*. *melitensis* strain in streptomycin-containing media until a streptomycin-resistant strain was isolated [8]. The resulting isolate, Rev. 1, was found to have reduced virulence and to protect against abortion in small ruminants. Much of what is known of the pathogenesis of *B*. *melitensis* infection in small ruminants comes from research on the efficacy of the Rev. 1 vaccine, conducted in the 1950s and ‘60s [8–11]. Considerable advances have recently been made in the field of brucellosis research, but most studies have been conducted in mouse models. Mice are highly resistant to *Brucella* infection [12]. Study of reproductive lesions, tissue colonization, and shedding of organism is best performed in a ruminant host, as these aspects of disease pathogenesis are difficult to extrapolate from mouse models. The utility of the small ruminant as a model for *B*. *abortus* [13–17] and *B*. *melitensis* infection [18–26] is well established, but challenges associated with working with large animals in biosafety level 3 containment facilities have limited the work done with virulent strains. Much of what is known of the pathogenesis of virulent *B*. *melitensis* in goats comes from findings published on unvaccinated control goats utilized in vaccination and challenge studies [27–31]. There is a critical need for further research on *B*. *melitensis* pathogenesis and host immune response to both virulent and vaccine strains of *B*. *melitensis* as few countries with endemic *B*. *melitensis* infection have successfully eradicated this pathogen [32].

The goal of the present research was to study the course of pathogenesis and immunity following *B*. *melitensis* infection in goats by evaluating disease caused by both the fully virulent *B*. *melitensis* strain 16M and the reduced virulence strain Rev. 1. Objectives were to (1) determine the clinical effects of infection by each strain, (2) evaluate tissue distribution and level of colonization resulting from infection, (3) quantify shedding of *B*. *melitensis* 16M and Rev. 1 in milk and uterine secretions, and (4) evaluate the humoral immune response triggered in response to infection, especially the stage of infection during which antibodies are first detected.

## Materials and methods

### Ethics statement

All experiments were approved by the Institutional Animal Care and Use Committee of Colorado State University, Fort Collins, Colorado, USA, under approval number 14-5114A.

### Animals and experimental design

Fifteen mixed-breed female goats (aged 2–9 years) were utilized in this study. The animals had not been previously *Brucella*-vaccinated and were seronegative prior to challenge. Estrous cycles of the does were synchronized using two injections, 10 days apart, of prostaglandin F2α (Lutalyse, Zoetis, Inc.), followed by mating to a fertile male. All animals were initially found to be pregnant by ultrasound examination; however, does 5, 7, and 11 subsequently suffered early fetal losses, as these goats showed no evidence of abortion and were not pregnant at necropsy. Animals were transferred to a biosafety level 3 containment facility at least one week prior to experimental challenge, where they were housed for the remainder of the study. All animals were fed a complete pelleted diet and hay daily with nutritional supplements added in late gestation. Animals were group-housed in rooms of adequate size (2–4 goats per room), and all stages of the study were conducted with consideration for animal welfare. Animals were monitored twice daily by either a veterinarian or veterinary student and any medical concerns promptly addressed. In addition to assessing behavior and appetite, rectal temperature and complete blood counts were utilized to assess health. This study was carried out over the course of 2 years with 5 goats challenged in March 2014 (group 1) and the remaining 10 animals challenged in January 2015 (groups 2–4). Challenge was conducted at either 11 (group 1) or 14 weeks (groups 2–4) of gestation. Since animals within each group were housed together, factors such as body size, presence of horns, and temperament were considered when assigning animals to groups 2–4 in the 2015 study. Animals were infected by instillation of 8 x 10^6^–8 x 10^7^ CFU of *B*. *melitensis* onto their conjunctiva (50 μl of inoculum per eye). The infection dose was determined by serial dilution of the inoculum in saline and standard plate count on brain heart infusion (BHI) agar plates. Nine animals (5 goats in 2014 and 4 goats in 2015) were challenged with virulent *B*. *melitensis* strain 16M (BEI Resources, Manassas, VA) (groups 1 and 2), and 4 animals were challenged with the attenuated vaccine strain *B*. *melitensis* Rev. 1 (Dr. Thomas Ficht, Texas A&M University) (group 3). The remaining two goats were kept as uninfected controls (group 4). Animals were monitored closely for clinical signs of infection, such as changes in mentation and elevation of body temperature. Temperature was measured either per rectum or via an iButton (Maxim Integrated, San Jose, CA), which were administered *per os* prior to infection of groups 2–4 and then subsequently recovered at necropsy from the reticulum.

### Sampling procedures

#### Samples from does

Blood samples were collected by jugular venipuncture from all animals prior to challenge and following challenge on days 3, 7, 14, 21, 28, 35, and at time of parturition. Blood samples were utilized for bacteriological culture (group 1 only) and assessment of humoral immune response. Aseptic technique was utilized during venipuncture to prevent contamination of samples for culture.

Approximately 10 ml of milk was collected on the day of parturition and over each of the subsequent 3 days. Vaginal swabs were collected on the day of parturition and at necropsy. All does were humanely euthanized 3 days following abortion or normal parturition by intravenous administration of pentobarbital. Maternal samples collected aseptically at necropsy for bacteriological determination included: spleen, liver, lung, mammary tissue, uterine caruncle, muscle, and lymphatic tissue (bronchial, mediastinal, hepatic, internal iliac, mesenteric, mandibular, parotid, retropharyngeal, prescapular, and supramammary lymph nodes).

#### Samples from kids

Fetuses were necropsied within 24 hours of abortion. Live kids were kept together with the group of does and allowed to nurse until euthanasia and necropsy at 3 days of age. Samples collected at necropsy included spleen, liver, lung, abomasum, abomasal contents, and rectal swab. Placental tissue was also collected for culture.

### Bacteriological tests

Culture of blood, milk, vaginal swabs, fetal rectal swabs, and all tissue samples was performed on selective medium agar formulated from *Brucella* medium base (Oxoid CM0169), *Brucella* selective supplement (Oxoid SR0083A), and 10% fetal bovine serum. All cultures were incubated at 37°C and 5% CO_2_. Typically, growth was observed in 3–4 days. *Brucella* cultures were identified based on colony morphology and growth characteristics. Isolates from Rev. 1 infected goats were sub-cultured onto BHI agar containing streptomycin (2.5 μg/ml) and incubated for 2 weeks at 37°C in air. This allowed for the differentiation of Rev. 1 and 16M strains since only Rev. 1 is capable of growing on streptomycin [33].

#### Blood culture

At each time point, 10 ml of whole blood were collected in EDTA for culture. These samples were diluted 1:1 in BHI broth containing amphotericin B (1 μg/ml), vancomycin (20 μg/ml), and sodium citrate (1% v/v) [34]. Of this dilution, 100 μl was plated directly on the *Brucella* selective media described above. The remaining enrichment culture was maintained at 37°C with weekly subcultures performed for 4 weeks. Plates were examined regularly for growth and declared negative if no colonies were observed after one month.

#### Milk

Milk samples were centrifuged (13,000 × g, 5 min) and cream and precipitate layers combined for culture. Serial dilutions of the cream/precipitate mixture were plated on *Brucella* selective media for semi-quantitative determination of shedding.

#### Vaginal and rectal swabs

Vaginal and fetal rectal swabs were directly streaked on *Brucella* selective media. In these samples shedding was assessed by the presence/absence of growth rather than quantification of bacterial colonies.

#### Tissues

Tissue samples weighing approximately 0.1 g were added to 2 ml snap-top tubes containing 900 μl of homogenization media (BHI broth with 10% glycerol) and stainless steel beads. Samples were homogenized using a mixer mill (Retsch Mixer Mill MM 400, Düsseldorf, Germany) for 5 min at a frequency of 25 Hz. Colonization (CFU/g) of tissues was determined by plating serial dilutions of tissue homogenate on *Brucella* selective media. The homogenate consisted of a 1:10 dilution of tissue and additional dilutions were subsequently made extending to 10^−8^.

### Serological tests

Sera were tested for antibodies to *Brucella* using the Card Test (National Veterinary Services Laboratories [NVSL], Ames, IA, USA) and the fluorescence polarization assay (FPA) (*Brucella abortus* Antibody Test Kit, Ellie LLC, Wisconsin, USA). The Card Test was performed with *B*. *abortus* antigen of 3% cell concentration according to the protocol provided by NVSL. Briefly, 30 μl of serum was mixed with an equal volume of antigen suspension and the degree of agglutination determined after a 4 min incubation period. The FPA assay was performed according to manufacturer’s instructions. Briefly, 10 μl of serum was added to sample diluent. Blank intensity readings were obtained prior to addition of 10 μl of antigen-fluorescein conjugate. A second intensity reading was subsequently taken with the instrument automatically subtracting the background reading and presenting a result in millipolarization units (mP). Samples with a ΔmP of <10 were considered test negative. Samples with a ΔmP >20 were considered test positive. Samples were classified as suspect with ΔmP values of 10–20. All FPA testing was performed at NVSL.

## Results

### Clinical results

#### Abortion

Pregnant does were infected with either the fully virulent *B*. *melitensis* strain 16M (groups 1 and 2) or the attenuated strain Rev. 1 (group 3). Differences between the groups are described in Table 1. Six of the 7 (86%) pregnant does infected with *B*. *melitensis* 16M aborted. The remaining animal (No. 9) delivered one dead and one live full-term kid. While the live kid (No. 9–2) was weak, it was determined not to be suffering and was not humanely euthanized until the standard 3 days post-parturition. Of the 4 animals infected with the attenuated *B*. *melitensis* strain Rev. 1, all delivered full-term kids. A gestation length of 21 weeks was used to define full-term birth. Two (29%) of the full-term kids from the Rev. 1 group, both from the same doe (No. 12), were dead at birth. Another kid (No. 10–1) was found dead 3 days after birth, however, whether death was due to *Brucella* infection is unknown. Three does (No. 5, 7, and 11) showed no evidence of abortion and were not pregnant at necropsy. These animals were initially determined to be pregnant by ultrasound observation prior to challenge. Uninfected controls remained healthy and delivered live, full-term kids.

**Table 1:** Effect of *B*. *melitensis* 16M and Rev. 1 on parturition in goats.

Group No.	Doe No.	Challenge Strain	Challenge Dose (CFU)	Week of Gestation at Challenge	Week of Gestation at Parturition	Kid No.	Fetus Condition	Isolation of *B*. *melitensis* at Parturition^d^			
								Fetus	Placenta	Vaginal Swab	Milk
1	1	16M	8 x 10^6^	11	17	1–1	Dead	+	+	+	+
						1–2	Dead	+			
	2	16M	8 x 10^6^	11	16	2–1	Dead	+	NC	+	+
						2–2	Dead	+			
	3	16M	8 x 10^6^	11	20	3–1	Dead	+	+	+	+
						3–2	Dead	+			
	4	16M	8 x 10^6^	11	17	4–1	Dead	+	+	+	+
	5	16M	8 x 10^6^	11	NP^a^			NA	NA	−	−
2	6	16M	8 x 10^6^	14	19	6–1	Dead	+	+	+	+
	7	16M	8 x 10^6^	14	NP^b^			NA	NA	−	NC
	8	16M	8 x 10^6^	14	19	8–1	Dead	+	NC	+	+
						8–2	Dead	+			
	9	16M	8 x 10^6^	14	21	9–1	Dead	+	+	+	+
						9–2	Live,Weak	−			
3	10	Rev. 1	8 x 10^7^	14	21	10–1	Live^c^	+	−	+	+
						10–2	Live	−			
	11	Rev. 1	8 x 10^7^	14	NP^b^			NA	NA	−	+
	12	Rev. 1	8 x 10^7^	14	22	12–1	Dead	+	+	+	+
						12–2	Dead	+			
	13	Rev. 1	8 x 10^7^	14	21	13–1	Live	−	−	−	+
						13–2	Live	−			
						13–3	Live	−			

^a^ NP = not pregnant at time of necropsy despite being declared pregnant on ultrasound at 6 weeks of gestation

^b^ NP = not pregnant at time of necropsy despite being declared pregnant on ultrasound at 12 weeks of gestation

^c^ Kid 10–1 was found dead 3 days after parturition

^d^ + = *B*. *melitensis* isolated, − = *B*. *melitensis* not isolated, NC = sample not collected, NA = not applicable

#### Body temperature

After a normal baseline was established, rectal temperature was recorded for goats in group 1 once daily for the first 2 weeks post-infection and then weekly until time of parturition. Normal temperature was defined as 100–104°F (37.8–40.0°C). Rectal temperatures remained within the normal range in these five 16M-infected goats throughout the course of the study. Attempts to use iButtons for measurement of core temperature every 4 hours in group 2 and 3 goats were unsuccessful. Despite applying lead sinkers to the iButtons to maintain them within the rumen-reticulum, the devices were passed by all but two of the goats and excreted in feces. Thus, no conclusions could be drawn regarding core body temperature in group 2 and 3 goats.

### Bacteriological results

#### Does – Tissue Colonization

*Brucella melitensis* was isolated from tissue samples or milk of all 13 infected does, indicating that experimental challenge was successful in all animals. The three does (No. 5, 7, and 11) that showed no evidence of abortion and were not pregnant at necropsy had low level tissue colonization. In the non-pregnant 16M-infected does, *Brucella* was isolated from the spleen and mandibular, retropharyngeal, and internal iliac lymph nodes of one animal (No. 5) and exclusively the hepatic lymph node of the other animal (No. 7). Colonization in these tissues was minimal (100–400 CFU/g of tissue). The non-pregnant Rev. 1-challenged doe (No. 11) showed no evidence of systemic infection. A small volume of serous fluid was collected from this animal’s teats at necropsy; however, and the “milk” was culture positive (30 CFU/ml).

**Fig 1:**
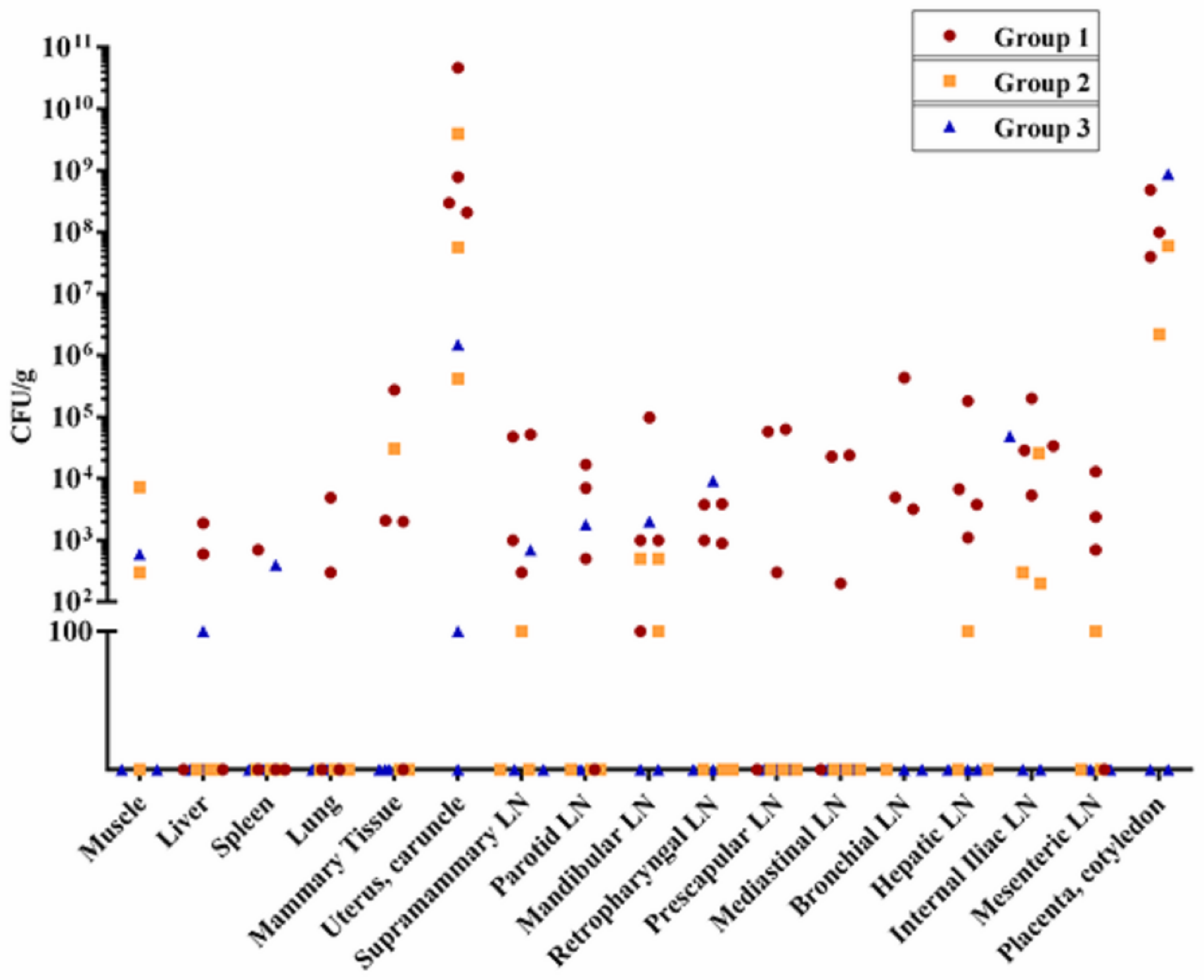
Level of tissue colonization by *B*. *melitensis* 16M and Rev. 1 in pregnant does. Colonization is measured in CFU of *B*. *melitensis* 16M (groups 1 and 2) or Rev. 1 (group 3) per gram of tissue. Limit of detection is 100 CFU.

A similar colonization pattern was observed in a pregnant Rev. 1-infected goat. *Brucella melitensis* was not cultured from any tissue from doe number 10 including the mammary gland and associated lymph nodes, uterus, and placenta, yet this doe exhibited low level excretion (10 CFU/ml) of Rev. 1 in milk on days 2 and 3 postpartum, shed organisms in vaginal secretions, and one kid showed low levels of colonization (Table 1). Likewise, another Rev.1-infected goat (No. 13) showed minimal colonization with just 100 CFU/g and 10 CFU/ml of *B*. *melitensis* isolated from uterine tissue and milk, respectively. The culture results from the final Rev. 1 infected animal (No. 12) show, however, that this strain of *B*. *melitensis* does have the capacity to cause severe generalized infection in some pregnant goats. *Brucella* was isolated from 9 different maternal tissues, placenta, and milk of this animal with high levels of colonization noted. A higher concentration of *B*. *melitensis* was present in the placenta of this animal than in any of the 16M-infected goats. All isolates from Rev. 1-challenged goats grew on streptomycin-containing media, confirming that no 16M contamination occurred in group 3 goats. Rate of *B*. *melitensis* isolation and tissue burden in pregnant animals is shown in Table 2 and Fig 1.

**Table 2:** Rate of isolation of *B*. *melitensis* 16M and Rev. 1 from tissues of pregnant does at necropsy.

Tissue	*B*. *melitensis* 16M Infected Animals	*B*. *melitensis* Rev. 1 Infected Animals
Muscle	2/3	1/3
Liver	2/7	1/3
Spleen	1/7	1/3
Lung	2/7	0/3
Mammary Tissue	4/7	0/3
Uterus	7/7	2/3
Supramammary LN	5/7	1/3
Parotid LN	3/7	1/3
Mandibular LN	7/7	1/3
Retropharyngeal LN	4/7	1/3
Prescapular LN	3/7	0/3
Mediastinal LN	3/7	0/3
Bronchial LN	3/4	0/2
Hepatic LN	5/7	0/3
Internal Iliac LN	7/7	1/3
Mesenteric LN	4/7	0/3
Placenta	5/5	1/3

**Fig 2:**
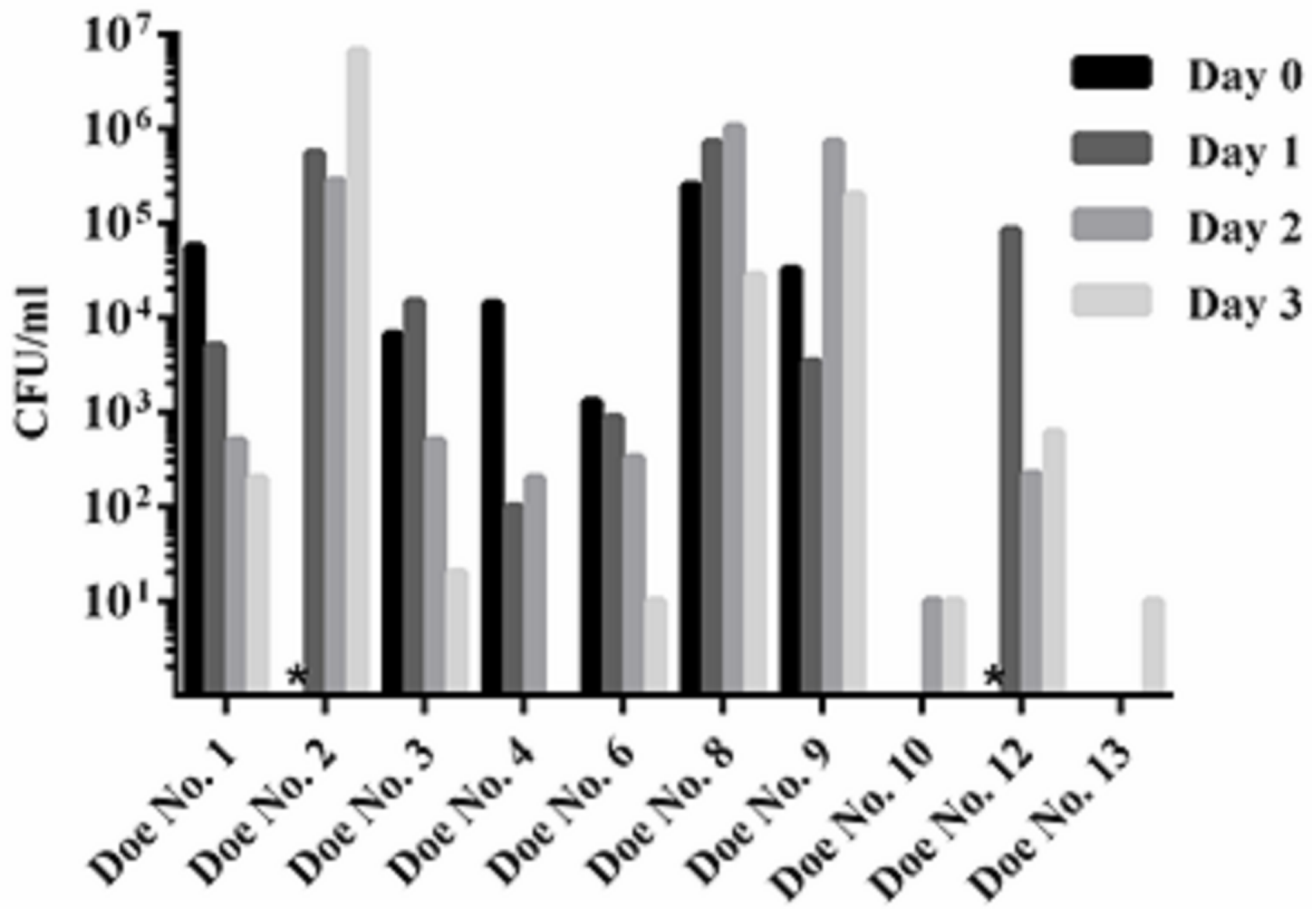
Shedding of *B*. *melitensis* 16M and Rev. 1 in milk. Shedding is measured in CFU of *B*. *melitensis* 16M (does 1–9) or Rev. 1 (does 10–13) per ml of milk cream/pellet. Limit of detection is 10 CFU. The absence of a bar indicates that *Brucella* was not isolated, except where an asterisk is placed to indicate that cultures were contaminated or not collected. Data from does 5, 7, and 11 are not included since these animals were not pregnant.

Generalized infection was present in all pregnant 16M-challenged goats, with rates of isolation varying from 4/16 to 15/15 tissues in the 7 goats sampled (Fig 2). In 16M-infected goats, *B*. *melitensis* displayed a preference for various tissues. Interestingly, organs such as liver, spleen and lung showed low levels of colonization. *Brucella* was more frequently isolated from lymph nodes, with all 7 pregnant goats showing colonization of the internal iliac lymph node. Lymph nodes of the head, which drained the site of inoculation, as well as the hepatic lymph node, the mesenteric lymph node, and the supramammary lymph node also showed high rates of colonization. Uterine and placental infection were noted in all pregnant16M-infected goats, and these two tissues were the most heavily colonized, with *B*. *melitensis* present at concentrations of 10^5^–10^10^ CFU/g of tissue. Concentration of organism in lymph node tissue was typically in the range of 10^2^–10^5^ CFU/g of tissue. Unexpectedly, muscle tissue (sampled from the appendicular skeleton, specific muscle group not identified) was positive in 2 of 3 pregnant 16M-challenged goats from which cultures were taken with tissue load similar to that in lymph nodes. While only performed in group 1 goats, all blood cultures were negative throughout the course of infection (cultures taken on days 3, 7, 14, and 28 post-infection and day 1 postpartum).

#### Does—Shedding

*Brucella melitensis* 16M was shed at high levels in milk of all pregnant animals challenged with this virulent strain (Fig 2). Three does were consistent high level shedders with *B*. *melitensis* present in milk at a concentration of 10^4^–10^7^ CFU/ml throughout the 4 days of sampling. The remaining 4 does showed a declining trend in the degree of shedding postpartum. In these animals *Brucella* was shed at a concentration of 10^2^–10^4^ CFU/ml in colostrum, but by day 4 numbers of organisms were nearing the limit of detection. Shedding in milk was minimal in all but one of the Rev. 1-infected goats. This doe (No. 12) excreted organism at levels similar to goats infected with *B*. *melitensis* 16M. Shedding in vaginal secretions occurred in all 16M-infected goats on both days 1 and 3 postpartum. In Rev. 1-infected does shedding was observed in 2/3 animals, but at lower levels.

#### Kids—Tissue colonization

A 92% fetal infection rate was observed among kids born to *B*. *melitensis* 16M-infected does (Table 3). Infection was systemic; organism was isolated from liver, spleen, lung, and abomasum in all infected kids. Tissue burden ranged from 103–10^9^ CFU/g of tissue with severely autolyzed fetuses typically having lower concentrations of *Brucella*.

**Table 3:** Isolation of *B*. *melitensis* 16M and Rev. 1 from fetal tissues at time of parturition.

Group No.	Kid No.	Fetus Condition	No. of samples containing *B*. *melitensis*	Tissue Burden (CFU/g)^b^					
				Liver	Spleen	Lung	Abomasum	Abomasal Contents	Rectal Swab
1	1–1	Dead	6/6	+ + + +	+ + + +	+ + + +	+ + + +	+ + + +	L
	1–2	Dead	6/6	+ +	+ + +	+ +	+ + +	+ + +	L
	2–1	Dead	6/6	+ + + +	+ + + +	+ + + +	+ + + +	+ + +	L
	2–2	Dead	6/6	+ +	+ + +	+ +	+ + +	+ +	L
	3–1	Dead	4/4	+ +	+ + +	+ +	+ +	NC	NC
	3–2	Dead	4/4	+ + +	+ + + +	+ + + +	+ + +	NC	NC
	4–1	Dead	6/6	+ +	+ +	+ +	+ +	+ +	L
2	6–1	Dead	6/6	+ +	+ + +	+ + +	+ +	+ + +	L
	8–1	Dead	6/6	+ + + +	+ + + +	+ + + +	+ + + +	+ + + +	L
	8–2	Dead	6/6	+ + + +	+ + + +	+ + + +	+ + +	+ + + +	L
	9–1	Dead	6/6	+ + +	+ + +	+ + +	+ + +	+ + + +	L
	9–2	Live,Weak	0/6	−	−	−	−	−	−
3	10–1	Live^a^	3/6	−	+	−	−	+	G
	10–2	Live	0/6	−	−	−	−	−	−
	12–1	Dead	6/6	+	+ +	+ +	+	+	G
	12–2	Dead	5/5	+ +	+ + +	+ + + +	+ + +	+ + +	NC
	13–1	Live	0/6	_	_	_	_	_	_
	13–2	Live	0/6	_	_	_	_	_	_
	13–3	Live	0/6	_	_	_	_	_	_

^a^ Kid 10–1 was found dead three days after parturition

^b^ + = 1 x 102–1 x 10^3^ CFU/g

+ + = 1 x 10^3^–1 x 10^5^ CFU/g

+ + + = 1 x 10^5^–1 x 10^7^ CFU/g

+ + + + = 1 x 10^7^–1 x 10^9^ CFU/g

− = *B*. *melitensis* not isolated, limit of detection = 1 x 10^2^ CFU/g

L = lawn

G = some growth

NC = sample not collected

Infection rate among kids born to *B*. *melitensis* Rev. 1-infected does was 43%. Doe number 12, which showed high levels of maternal, uterine, and mammary infection gave birth to two dead, heavily infected kids. Low level infection was also observed in one kid born to doe number 10. *Brucella melitensis* Rev. 1 was isolated from the spleen and abomasal fluid of this kid at concentrations of 100 and 900 CFU/g, respectively. The animal was also potentially shedding organism in feces since *Brucella* was cultured from a rectal swab.

### Humoral immune response

Anti-*Brucella* antibodies were detected in all 13 challenged does by the Card Test (Table 4). Seroconversion was delayed in the non-pregnant 16M-challenged animals, occurring at 61 and 70 days post-infection. In pregnant animals challenged with virulent *B*. *melitensis*, median time to seroconversion as determined by Card Test was 21 days (range 14–64 days); however, 2 animals did not seroconvert until after abortion. All *B*. *melitensis* Rev. 1-challenged does were seropositive by 4 weeks post-infection as determined by Card Test; titers were transient with only the heavily infected doe (No. 12) still seropositive at time of parturition. The two uninfected controls remained seronegative throughout the duration of the study. The FPA was found to be less sensitive than the Card Test (Table 4).

**Table 4:** Time to seroconversion in *B*. *melitensis* 16M- and Rev. 1-infected does determined by Card Test and FPA.

Doe No.	Treatment Group	Days until Seroconversion^a^ Card Test	Days until Seroconversion^a^ FPA
1	16M	21	21
2	16M	35	Neg
3	16M	64	64
4	16M	21	Neg
5	16M	70	Neg
6	16M	14	37
7	16M	60	Neg
8	16M	14	28
9	16M	28	Neg
10	Rev. 1	28	Neg
11	Rev. 1	28	28
12	Rev. 1	14	14
13	Rev. 1	28	28
14	Control	Neg	Neg
15	Control	Neg	Neg

^a^Weak positive (Card Test) and suspect (FPA) results were considered positive when determining time of seroconversion.

## Discussion

The present study provides a thorough assessment of the clinical effects resulting from *B*. *melitensis* infection in goats, a natural host. Rates of abortion and fetal death, distribution of organism in tissues, and levels of shedding were investigated. By characterizing these aspects of disease pathogenesis in goats infected with both *B*. *melitensis* strain 16M and strain Rev. 1, the study provides a starting point from which differences in virulence factors or host immune response to infection can be compared between the two strains. Body temperature and humoral immune response were also evaluated as potential measurable parameters suggestive of *Brucella* infection prior to shedding.

An obvious component of brucellosis control is the identification of infected animals. This is a critical first step in test and slaughter programs; however, even in resource poor countries where test and slaughter cannot be implemented, identification of infected animals allows herders to take proper protective measures to reduce spread of disease. Optimally, diagnosis of *Brucella* infection would be made prior to parturition before massive shedding of organism occurs in vaginal fluid, fetal tissues, and milk. In the present study, infection was inapparent for a minimum of 2–3 weeks. In two animals infected with *B*. *melitensis* 16M, we were even uncertain if the inoculum was successfully administered until time of abortion and tissue culture. In these animals there was no clear indication of infection based on clinical signs, bacteriology, or serology until after abortion occurred, underscoring the challenges associated with diagnosis of *Brucella* infection. No changes in behavior, appetite, or elevation in body temperature were observed post-infection. This agrees with previous reports which have found no systemic temperature response to conjunctival infection with Rev. 1 or virulent *B*. *melitensis* [26, 35] or only a transient 48 hr period of slight fever after subcutaneous inoculation with *B*. *melitensis* 16M [18].

*Brucella*-specific diagnostic tests also did not provide evidence of infection in all animals prior to abortion, and blood cultures failed to detect *Brucella* in any of the animals sampled. The inability to detect bacteremia may have been due to insufficient sampling volume or frequency, as tissue culture results at necropsy indicated that bacteremia had obviously occurred. Previous work has shown variable rates of potentially episodic bacteremia. Cheville et al. found 50% of goats to be bacteremic 3 days post-infection with 16M [18]. Similar rates of bacteremia (25–50%) have also been reported 2–3 weeks post-infection [8]. While circulating antibodies were more easily detected than brucellae in the present study, diagnosis of infection by serology in all animals prior to abortion was still not possible. Only 55% of pregnant 16M-infected does had circulating anti-*Brucella* antibodies detectable by the Card Test by 3–4 weeks post-infection, and 2 animals did not seroconvert until after abortion. Previously reported seroconversion rates in small ruminants conjunctively infected with Rev. 1 or 16M are 90–100% seroconversion by 30 days post-infection [23, 26, 36]. Delay in seroconversion until after abortion, however, has previously been noted in cattle [37, 38]. Extensive investigation of abortions in New Zealand during the 1970’s indicated that in 13% of cows, *Brucella* infection could only be diagnosed by culture from tissues.

There have been recent advances in *Brucella* serological testing. The FPA has been increasingly applied in diagnosis of bovine brucellosis due to its high sensitivity and specificity, ability to distinguish infected and vaccinated animals, and its utility in a field setting. Studies in small ruminants have reported variable results. Nielsen reports that the sensitivity and specificity of a FPA based on a *B*. *abortus* antigen is 95% and 99% respectively [39], while Ramierez-Pfeiffer reports sensitivity and specificity in small ruminants is 79% and 89% respectively with a *B*. *abortus* antigen and 86% and 92% with a *B*. *melitensis* antigen [40]. The FPA test kit utilized in the present study is the sole USDA-approved FPA for diagnosis of brucellosis in the U.S, and it utilizes a *B*. *abortus* antigen. To the authors’ knowledge this FPA kit has not been evaluated in goats. In the 15 animals tested in the present study, the 3% Card Test was 100% sensitive and 100% specific. Specificity of the FPA was 100%; however, sensitivity was only 75% among Rev. 1-infected goats and 44% among 16M-infected goats. This suggests further optimization of the USDA-approved FPA test kit is necessary before this assay is used in field testing of goats. These findings also challenge previous reports, which indicate that the FPA can distinguish infected from vaccinated animals, at least in cattle [41].

While humoral immune response and early clinical signs were ambiguous in many of the *B*. *melitensis*-infected goats, abortion was found to be a clear indicator of infection. Among *B*. *melitensis* 16M-infected does, an 86% abortion rate was observed, with 92% of offspring infected and dead at birth. This rate of abortion is within the range cited in recent literature. Typically, 70–100% of pregnant goats experimentally infected with *B*. *melitensis* abort [22, 29, 42]. While all Rev. 1-infected animals had full-term parturitions, infection was not without clinical effects. Two kids, both from the same doe, were dead at birth and another kid was found dead three days later. It is well known that Rev. 1 retains some virulence in goats and vaccination during pregnancy is not recommended [26].

*Brucella melitensis* 16M was found to produce generalized infection in pregnant does, with brucellae recoverable from as many as 15 different tissue types in a single animal. Predictably, the uterus and placenta showed both the highest rates of colonization (100%) and the highest tissue burdens (up to 5 x 10^10^ and 5 x 10^8^ CFU/g in the uterus and placenta, respectively). These rates of colonization are similar to those previously reported in goats infected with *B*. *abortus*; the average placentome concentration of *B*. *abortus* was reported to be 2 x 10^8^ CFU/g [14].

Bacteriological culture of tissues from pregnant 16M-infected does also yielded unexpected results with important public health implications. Muscle tissue (from the appendicular skeleton, specific muscle group not identified) was cultured from 3 does with the initial intension of simply obtaining bacteriological confirmation of the safety of muscle tissue. Generally, handling and consumption of raw or undercooked organ meat is considered a potential health risk, but muscle meat has been assumed safe [43]. In 2 of the 3 animals from which muscle cultures were performed, however, *Brucella* was isolated at concentrations comparable to that in organs and lymph nodes (10^2^–10^4^ CFU/g). This was repeatable and other tissues collected from these two animals were culture negative, indicating that contamination of culture media and equipment was unlikely. Due to small sample size in the present study, infection of muscle tissue should be further investigated. To the author’s knowledge this is the first report of the isolation of *Brucella* from muscle tissue in ruminants. In humans *Brucella*-associated myositis has been reported and may be under diagnosed [44, 45].

In comparison to muscle tissue, milk from *Brucella* infected animals is a well-known public health risk. The present study reinforces this health risk. We report shedding in 100% of post-parturient 16M-infected does; shedding was at the level of 10^4^–10^7^ CFU/ml of milk in approximately half of the goats sampled. While samples were concentrated to a degree by enriching for cream and pellet layers, an 8 oz (237 ml) serving of unpasteurized milk would contain over a million infectious doses.

Among the 16M infected does, approximately half of the does were consistent, high level shedders and half showed a declining trend in shedding over the 4 sampling days. Without continued monitoring of *Brucella* concentrations in milk, it is difficult to infer whether these groups would hold throughout the lactation period. In sheep experimentally infected with *B*. *melitensis*, shedding in milk persisted for at least three reproductive cycles post-infection [25]. In that study, the percent of sheep shedding *Brucella* in milk was 79%, 64%, and 38% over the first, second, and third reproductive cycles post-infection, respectively. A recent study on water buffalo naturally infected with *B*. *abortus* indicates that while most animals intermittently shed brucellae at low levels in milk, 16% of infected animals can be classified as “superspreaders” since they consistently shed high levels of organism [46]. These animals are of considerable public health risk, as well as the primary source of disease in the herd. Culling of superspreaders was found to halt disease transmission within a herd. The present study suggests that similar groups of high and low level shedders may exist among *B*. *melitensis*-infected goats.

### Study limitations

The objective of the present study was a descriptive analysis of *B*. *melitensis* pathogenesis in goats. Due to small sample size within each group, no attempt was made to conduct statistical analysis of differences between clinical effects, tissue burden, and shedding in 16M- and Rev. 1- infected goats. Sample size was limited due to the space limitations and financial constraints associated with large animal work in a biosafety level 3 facility. An additional limitation was that the culture methods utilized in the present study had a 100 CFU and 10 CFU limit of detection from tissue and milk, respectively. In addition, only a small section of tissue was collected from each organ sampled, and, thus, focal infection within an organ could be missed.

## Conclusions

The clinical effects, pathogenesis, and shedding of two strains of *B*. *melitensis* in goats are reported in the present study. Infection of goats with *B*. *melitensis* 16M resulted in an 86% abortion rate with all but one kid exhibiting generalized infection leading to fetal death (Table 5). *Brucella melitensis* 16M disseminated widely in pregnant does post-infection with none of the 15 sampled tissues spared from colonization. Importantly, we report the first isolation of *B*. *melitensis* from muscle tissue in ruminants with colonization at levels of 10^2^–10^4^ CFU/g. Thus, muscle meat should potentially be added to the list of foodborne sources of *Brucella* infection in humans. For comparison, levels of shedding in milk reached levels of 10^4^–10^7^ CFU/ml in several goats, while placental and fetal tissue contained up to 10^9^ CFU/g. Pathogenesis of Rev. 1 infection was variable with two pregnant does showing minimal colonization and birth of full-term healthy kids and one doe exhibiting tissue colonization and clinical signs similar to those in animals infected with fully virulent 16M. Diagnosis of infection prior to parturition would greatly decrease disease transmission events. Unfortunately, *Brucella*-specific diagnostic tests including blood culture and serological assay were unable to diagnose infection in all animals prior to parturition. Postpartum identification of animals shedding high levels of organism in milk may benefit disease control efforts. Selective culling of high-level shedders may be an effective and feasible alternative to a comprehensive test and slaughter program.

**Table 5:** Summary of results.

	*B*. *melitensis 16M Infected Animals*^a^	*B*. *melitensis Rev*. *1 Infected Animals*^a^
1. Frequency of Abortion	*86%*	*0%*
2. Percent of Kids Born Dead	*92%*	*29%*
3. Fetal Infection Rate		
a) Any degree of infection	*92%*	*43%*
b) Generalized infection^b^	*92%*	*29%*
4. Maternal Infection Rate^c^	*100%*	*66%*
a) Any degree of infection	*100%*	*33%*
b) Generalized infection^b^		
5. Shedding in Vaginal Secretions	*100%*	*66%*
6. Shedding in Milk	*100%*	*100%*
a) Any degree of shedding		
b) Consistent high level of shedding	*43%*	*33%*
7. Seroconversion Prior to Parturition	*71%*	*100%*

^**a**^ Only data from pregnant animals are included in the table.

^b^ Generalized infection is defined by recovery of *B*. *melitensis* from three or more tissues.

^c^ Maternal infection is defined by recovery of *B*. *melitensis* from any maternal sample excluding fluid secretions such as vaginal fluid and milk.

## Supporting information

S1 File Raw data files. File A in S1. Goat no. 1–5 serology results. File B in S1. Goat no. 6–15 sample groups. File C in S1. Goat no. 6–15 serology results. File D in S1. USDA FPA results. File E in S1. Goat no. 1–5 tissue culture and body temperature results. File F in S1. Goat no. 6–15 tissue culture results. File G in S1. Goat no. 9–11 iButton temperature results.
(ZIP)
