# Supplementary material for: Evaluation of shedding, tissue burdens, and humoral immune response in goats after experimental challenge with the virulent Brucella melitensis strain 16M and the reduced virulence vaccine strain Rev. 1
Source: PLoS One. 2017 Oct 13;12(10):e0185823. doi: 10.1371/journal.pone.0185823 (PMC5640220; doi:10.1371/journal.pone.0185823)
Supplement: S1 File — File A in S1. Goat no. 1–5 serology results. File B in S1. Goat no. 6–15 sample groups. File C in S1. Goat no. 6–15 serology results. File D in S1. USDA FPA results. File E in S1. Goat no. 1–5 tissue culture and body temperature results. File F in S1. Goat no. 6–15 tissue culture results. File G in S1. Goat no. 9–11 iButton temperature results. (ZIP) [file pone.0185823.s001.zip › S1 File B.docx]

Lab Notebook – Spring 2015 Goat Project Wednesday, January 7, 2015

1. Ultrasounded goats today to confirm pregnancy (should be 88 days). Also estimated age of goats:

| **Goat Number** | **Breed** | **Pregnancy Status** | **Approximate Age** |
| --- | --- | --- | --- |
| 1588 | White Saanan, no horns | P, Twins, <90 days? | 5+ |
| 29 | Alpine | P | 3+ |
| “Tiny” | Alpine | O | <1 |
| COCN45 | Nubian | P, 2-3 fetuses | 4+ |
| 01131 | Boer | P | 4+ |
| 06 | Boer | P | 5+ |
| 01125 | Boer | P, 1 fetus | 2 |
| 01144 | Boer | P, 2+ fetuses | 3 |
| 0624 | Tan Saanan, w/ horns | P | 5+ |
| 01170 | Brown Lamancha | P | 3 ½ |
| 32 | Red Boer | P | 3 ½ |

*Total sample size is now ten. The young alpine goat is most likely open and will not be used.

1. Moved goats into ADL with goats group by behavioral compatibility:

Goats in *B. melitensis* 16M room: **Goat No. 6-9**

- 01131
- 01125
- 01144
- 0624

Goats in *B. melitensis* Rev1 room: **Goat No. 10-13**

- 29
- COCN45
- 32
- 1588

Goats in control (uninfected) room: **Goat No. 14-15**

- 01170
- 06
